# Wenn Zugehörige häuslich pflegen: Zusätzliche Belastung oder persönlicher Zugewinn für Freund*in, Nachbar*in und Bekannte*n? – Ergebnisse einer explorativen Querschnittstudie

**DOI:** 10.1055/a-2736-6425

**Published:** 2026-02-02

**Authors:** Natascha Lauer, Maria Heckel, Sophia Bösl, Johanna Schmidt, André Kratzer, Elmar Gräßel, Anna Pendergrass

**Affiliations:** 1 Zentrum für Medizinische Versorgungsforschung, Psychiatrische und Psychotherapeutische Klinik, Uniklinikum Erlangen, Friedrich-Alexander-Universität Erlangen-Nürnberg (FAU), Erlangen, Germany; 2 Palliativmedizinische Abteilung, CCC Erlangen–EMN, Uniklinikum Erlangen, Friedrich-Alexander-Universität Erlangen-Nürnberg (FAU), Erlangen, Germany

**Keywords:** häusliche Pflege, informell pflegende Person, pflegende Zugehörige, pflegende Angehörige, Belastung, Zugewinne, informal care, informal caregivers, non-kin caregivers, family caregivers, burden, benefits

## Abstract

**Hintergrund:**

Obwohl der Anteil an Zugehörigen, die in der Nachbarschaft, dem Freundes- oder Bekanntenkreis häuslich pflegen, steigt, fehlt es an Untersuchungen, die über die allgemeinen Charakteristika dieser Subgruppe hinausgehen. Ziel dieser explorativen Studie ist es, die Population der pflegenden Zugehörigen erstmals tiefergehend zu untersuchen und Unterschiede zu pflegenden Angehörigen insbesondere hinsichtlich der Belastung und dem persönlichen Zugewinn durch die häusliche Pflege aufzuzeigen.

**Methodik:**

Die Daten stammen aus einer repräsentativen Stichprobe (N=2927) pflegender Zu- und Angehöriger gesetzlich versicherter Pflegebedürftiger aus Bayern, die im Rahmen einer Querschnittsstudie in Kooperation mit dem MD Bayern befragt wurden. Für die pflegenden Zugehörigen (N=71) wurden die allgemeinen Charakteristika, die Belastungen und die Zugewinne durch die Pflege untersucht. Der Hauptfokus lag auf vergleichenden Analysen mit den pflegenden Angehörigen (N=2856) mittels χ2 -Tests und t-Tests für unabhängige Stichproben bzw. entsprechenden non-parametrischen Verfahren.

**Ergebnisse:**

Pflegende Zu- und Angehörige unterschieden sich nicht hinsichtlich der allgemeinen Charakteristika. Trotz gleicher objektiver Belastung (t(74,45)=1,80, p=0,077) nahmen die Zugehörigen die Pflegesituation als signifikant weniger belastend wahr (t(72,56)=2,57, p=0,012), erlebten signifikant mehr Zugewinne durch die häusliche Pflege (t(2925)=−3,37, p<0,001) und schätzten ihre psychische Gesundheit signifikant positiver ein (t(72,84)=−2,52, p=0,014).

**Schlussfolgerung:**

Pflegende Zugehörige leiden seltener an hohen subjektiven Belastungen und profitieren darüber hinaus häufig persönlich von der Unterstützung, Betreuung oder Pflege einer Person aus der Nachbarschaft, dem Freundes- bzw. Bekanntenkreis. Sie stellen somit eine Ressource dar, die in Zukunft verstärkt dazu beitragen könnte, die häusliche Pflege zu stärken.

## Abstract (en)

**Background:**

Although the proportion of non-kin caregivers providing home care for friends, neighbours, and acquaintances is steadily increasing, there is a lack of research that goes beyond the general characteristics of this subgroup. The aim of this exploratory study is to provide the first in-depth examination of the non-kin caregiver population and highlight differences between non-kin and family caregivers in terms of the burden and perceived benefits of informal care.

**Methods:**

The data are based on a representative sample (n=2927) of non-kin and family caregivers of statutorily insured care recipients, surveyed in a cross-sectional study in cooperation with MD Bayern. The general characteristics, burden, and perceived benefits of non-kin caregivers (n=71) were examined. The main focus was on comparative analyses with family caregivers (n=2856) using χ²-tests and independent samples t-tests, or corresponding non-parametric tests.

**Results:**

Non-kin and family caregivers did not differ in terms of general characteristics. Despite having the same objective burden (t(74.45)=1.80, p=0.077), non-kin caregivers perceived the caregiving situation as significantly less burdensome (t(72.56)=2.57, p=0.012), experienced significantly more benefits from informal care (t(2925)=−3.37, p<0.001), and rated their mental health significantly more positively (t(72.84)=−2.52, p=0.014).

**Conclusion:**

Non-kin caregivers are less likely to experience high levels of subjective burden and often derive greater personal benefit from the support, care, or assistance of someone from their neighborhood, social circle, or acquaintances. As such, they represent a valuable resource that could help strengthen informal care in the future.

## Einleitung

Derzeit werden 80 Prozent der ca. fünf Millionen pflegebedürftigen Personen (pP) in Deutschland im häuslichen Umfeld durch pflegende An- (pA) und Zugehörige (pZ) versorgt 1 . Als pA werden Personen verstanden, die mit der pP im nahen oder entfernten Verwandtschaftsverhältnis stehen oder im Verwandtschaftsnetz solidarisch agieren (z.B. nicht verheiratete Partner*innen, Schwager/Schwägerin) 2 . Davon abzugrenzen sind nicht-verwandte pZ, die mit der pP in einer „selbstgewählten sozialen Beziehung“ 2 stehen (Freund*innen, Nachbar*innen und andere nicht-verwandte Personen wie Bekannte 3 ).

Fast 35% der Personen ab 65 Jahren 4 und fast 45% der Hochaltrigen ab 80 Jahren 5 leben alleine. Wenn pP ein räumlich verfügbares Netzwerk an verwandten Personen fehlt, greifen sie verstärkt auf nicht-verwandte Personen zur Bewältigung ihres Alltags zurück 6 7 8 . So verbringen knapp 41% der älteren Menschen in Deutschland häufig und 37% manchmal Zeit mit Zugehörigen 5 . Somit tragen pZ neben pA zum Verbleib der pP im häuslichen Umfeld bei 9 .

Die lange Zeit fehlende explizite definitorische Nennung der pZ führte dazu, dass informell pflegende Personen größtenteils als pA verstanden wurden und pZ als Subgruppe in der Forschungsliteratur vernachlässigt worden sind 10 . Die Untersuchung der pZ wird jedoch aufgrund der sich erhöhenden Diversität des helfenden Netzwerkes mit dem Alter immer zentraler 8 . Heute erhalten bereits 2,1% der pP in Deutschland Unterstützung oder Pflege durch pZ 11 .

### Allgemeine Charakteristika

Die wenige Literatur zu pZ fokussierte v.a. allgemeine Charakteristika der pZ 6 12 : Sie sind vorwiegend weiblich 13 , häufig unverheiratet 14 15 , zeigen ein höheres Alter 13 16 und höheres Bildungsniveau auf und sind seltener in Vollzeitbeschäftigung erwerbstätig 16 . Sie leisten eher instrumentelle, emotionale und informationelle Unterstützung. Seltener wird mittels körpernaher Tätigkeiten unterstützt 6 .

### Belastung durch die häusliche Pflege

Als objektive Belastung wird im Wesentlichen der quantifizierbare Aufwand definiert, der durch die häusliche Pflege entsteht (z.B. der Verlust an Freizeit 17 ). Unter subjektivem Belastungserleben wird dagegen ein völlig anderes, komplexes Konstrukt verstanden, das von weiteren psychosozialen Faktoren bestimmt wird (z.B. vorhandene soziale Unterstützung oder Copingstrategien) 18 . Während die hohe objektive und subjektive Belastung durch die Übernahme von unterstützenden und pflegerischen Tätigkeiten bei pA national und international bestätigt wurde 19 20 21 , ist sie für pZ kaum erforscht. Es konnte eine objektive zeitliche Belastung von bis zu sechs Stunden pro Woche für pZ ermittelt werden 13 , die subjektive Belastung wurde bisher nicht quantitativ untersucht. Erste qualitative Hinweise zeigen, dass pZ als Hauptpflegepersonen auch von Überlastungserleben berichten 14 22 23 .

### Zugewinne durch die häusliche Pflege

Die Zugewinne durch die häusliche Pflege, wie z.B. das Erlernen neuer Fertigkeiten oder sich wertgeschätzt fühlen 24 sowie deren „Puffereffekte“ auf die negativen gesundheitlichen Konsequenzen der häuslichen Pflege, wurden für pA quantitativ gezeigt 25 , jedoch noch nicht für pZ. Erste Hinweise eines solchen Effektes für pZ zeigt eine qualitative Studie auf, wonach die Pflege einer pP auch von zahlreichen positiven Aspekten begleitet wurde 22 .

Das Ziel der vorliegenden Studie ist es, mittels einer umfangreichen explorativ-hypothesengenerierenden Untersuchung eine Grundlage zu schaffen, um die aufgezeigte Forschungslücke zu schließen. Im Zentrum steht der Vergleich zwischen pZ und pA bezüglich der objektiven und der subjektiven Belastungen sowie des Zugewinnerlebens durch die häusliche Pflege.

## Methodik

### Design und Stichprobe

Die Daten wurden im Rahmen der Querschnittsstudie „Progression in home care (ProCare) – Befragung (Phase I)“ generiert. 500 Pflegegutachter*innen des Medizinischen Dienstes Bayern verteilten zwischen September und Dezember 2022 insgesamt 25 000 Fragebögen an pZ und pA in Bayern, die eine gesetzlich pflegeversicherte pP pflegten. Die Rücklaufquote der anonymen Befragung betrug 11,8% (2941 Fragebögen). Befragte mit mehr als 50% fehlenden Werten ( *n* =7), deren pP verstorben ( *n* =5), in vollstationäre Pflege wechselten ( *n* =1) oder den Fragebogen selbst ausfüllten ( *n* =1), wurden ausgeschlossen. Die finale Analysestichprobe umfasste *N* =2927 (11,7%) Teilnehmende. Mit der Fragebogenrücksendung stimmten die Teilnehmenden der anonymisierten Verwendung ihrer Angaben zu. Die Studie fand unter Zustimmung der Ethikkommission der Friedrich-Alexander-Universität Erlangen-Nürnberg statt (Ref. 20-220_2-B) und wurde im ISRCTN-Register registriert (ISRCTN13390923).

### Einschlusskriterien

Teilnehmende mussten deutschsprachig und die Hauptpflegeperson einer gesetzlich pflegeversicherten pP mit Hauptwohnsitz in Bayern sein, für die die erstmalige Beantragung einer Einstufung des Pflegegrads oder ein Antrag auf Höherstufung vorlag.

### Stichprobencharakteristika

Die soziodemographischen Variablen sind in Tab. 1 aufgeführt.

**Table:** **Tab. 1** Allgemeine Stichprobencharakteristika.

Variable	Zugehörige *N=* 71 *n* (%) / *M* ± *SD* ( *Spannweite* )	Angehörige *N=* 2856 *n* (%) / *M* ± *SD* ( *Spannweite* )	*p* ^1^
**Pflegende Person**			
Alter (in Jahren)	62,7±13,8 (19–91)	60,4±13,0 (17–96)	0,129
Geschlecht (weiblich)	58 (81,7)	2134 (74,7)	0,251 ^ 2^
Bildungsgrad			
Kein Schulabschluss	1 (1,4)	30 (1,1)	0,552 ^ 2^
Hauptschule	20 (28,2)	914 (32,0)	
Realschule/Mittlere Reife	27 (38,0)	1174 (41,1)	
(Fach-)Abitur/Fachhochschulreife	12 (16,9)	334 (11,7)	
(Fach-)Hochschulabschluss	11 (15,5)	404 (14,1)	
Erwerbstätigkeit (Ja)	31 (43,7)	1497 (52,4)	0,145
Migrationshintergrund (Ja)	4 (5,6)	379 (13,3)	0,059
Hauptpflegeperson (Ja)	57 (80,3)	1612 (56,4)	0,169
**Gepflegte Person**			
Alter (in Jahren)	77,3±13,4 (34–98)	75,6±20,3 (0–102)	0,674 ^ 3^
Geschlecht (weiblich)	43 (60,6)	1702 (59,6)	0,869
Demenzerkrankung (Ja)	18 (25,4)	841 (29,4)	0,454
Pflegegrad			
0	20 (28,2)	711 (24,9)	0,746
1	12 (16,9)	383 (13,4)	
2	20 (28,2)	984 (34,5)	
3	14 (19,7)	534 (18,7)	
4	5 (7,0)	244 (8,6)	
**Pflegesituation**			
Zusammenleben (Ja)	19 (26,8)	1612 (56,4)	**<0,001**
Pflege weiterer Personen (Ja)	16 (22,5)	495 (17,3)	0,254
ADLs (Stunden/Tag)	2,4±1,6 (0,4–7,5)	2,7±2,0 (0,7–15,6)	0,441 ^ 3^
IADLs (Stunden/Tag)	3,4±2,2 (0,5–12,0)	3,6±2,4 (0,0–16,1)	0,451 ^ 3^
Beaufsichtigung (Stunden/Tag)	3,4±3,2 (0,5–13,6)	3,9±3,5 (0,1–16,0)	0,149 ^ 3^

**Anmerkungen.** ADLs=Aktivitäten des täglichen Lebens (z. B. Hilfe beim Ankleiden, Spannweite 0–17 Stunden/Tag); IADLs=Instrumentelle Aktivitäten des täglichen Lebens (z. B. Hilfe beim Einkaufen, Spannweite 0-17 Stunden/Tag); Beaufsichtigung (z. B. Verhindern von Gefahrensituationen, Spannweite 0–17 Stunden/Tag). Signifikante *p* -Werte sind in Fettdruck dargestellt;

^1^ Die dargestellte *p* -Werte sind vor dem Hintergrund des explorativen Charakters der Studie zu interpretieren;

^2^ Verwendung des exakten Tests nach Fisher, da ein oder mehrere Zellen eine erwartete Zellhäufigkeit von weniger als 5 Fällen aufwiesen;

^3^ Verwendung des nichtparametrischen Mann-Whitney-U-Tests, da extreme Ausreißer (>2,5-facher Interquartilsabstand) vorlagen.

### Instrumente

Der deutschsprachige Selbstauskunftsfragebogen wurde im Paper-Pencil-Format ausgefüllt.

Die **objektive Belastung** durch die häusliche Pflege, erfasst als Anzahl der *Stunden der Pflegeleistungen* pro Tag, setzt sich zusammen aus den *Aktivitäten des täglichen Lebens* (ADLs, z. B. Hilfe beim Anziehen), *Instrumentellen Aktivitäten des täglichen Lebens* (IADLs, z. B. Zubereitung von Mahlzeiten) und der *Beaufsichtigung* (z. B. Vermeiden von gefährlichen Situationen), angelehnt an den *Resource Utilization in Dementia Questionnaire* 26 .

Zur Erfassung des **subjektiven Belastungserlebens** wurde die validierte *Kurzversion der Häuslichen Pflegeskala (HPS-k)* 18 27 und zur Erhebung der **erlebten Zugewinne** durch die häusliche Pflege wurde die validierte *Benefits of Being a Caregiver Scale (BBCS)* 24 verwendet. Diese werden in Tab. 2 näher erläutert.

**Table:** **Tab. 2** Spezifisches Belastungs- und Zugewinnerleben von pflegenden Zu- und Angehörigen.

Variable	Variablen-nummer	Zugehörige ( *N=* 71)	Angehörige ( *N=* 2856)	*p* ^1^	Effektstärke ^2^
**Subjektive Belastung** (HPS-k), *M* ± *SD*					
* ... Durch die Unterstützung/durch die Pflege hat die Zufriedenheit in meinem Leben gelitten.*	1	1,1±1,0	1,7±1,0	**<0,001**	**0,60**
* ...Ich fühle mich oft körperlich erschöpft.*	2	1,7±1,0	2,0±0,9	**0,029** ^3^	**0,30**
* * ... Ich habe hin und wieder den Wunsch, aus meiner Situation „auszubrechen“.	3	1,6±1,2	1,9±1,0	0,064 ^3^	
* ... Ich empfinde mich manchmal nicht mehr richtig als „ich selbst“.*	4	1,3±1,2	1,6±1,0	**0,034**	**0,26**
* * ... Mein Lebensstandard hat sich durch die Unterstützung/Pflege verringert.	5	1,4±1,1	1,6±1,0	0,175	
* ... Durch die Unterstützung/Pflege wird meine Gesundheit angegriffen.*	6	1,2±1,0	1,5±1,0	**0,012**	**0,31**
* * ... Die Unterstützung/Pflege kostet viel von meiner eigenen Kraft.	7	1,9±1,0	2,1±0,9	0,051 ^3^	
* * ... Ich fühle mich „hin- und hergerissen“ zwischen den Anforderungen meiner Umgebung (z. B. Familie) und den Anforderungen durch die Unterstützung/Pflege.	8	1,6±1,1	1,9±1,0	0,065 ^3^	
* * ... Ich sorge mich aufgrund der Unterstützung, die ich leiste/aufgrund der Pflege um meine Zukunft.	9	1,2±1,2	1,4±1,1	0,123	
* * ... Wegen der Unterstützung/Pflege leidet meine Beziehung zu Familienangehörigen, Verwandten, Freund*innen und Bekannten.	10	1,3±1,1	1,6±1,1	0,069	
**Erlebte Zugewinne** (BBCS), *M* ± *SD*					
Durch die Unterstützung/Pflege meines/e Angehörigen/Bekannten...					
* * ... habe ich mich selbst besser kennengelernt.	1	1,9±1,3	1,8±1,2	0,294	
* ... konnte ich die Beziehung zu ihm/ihr verbessern.*	2	2,6±1,3	1,9±1,2	**<0,001**	**0,57**
* * ... habe ich gelernt, meine Zeit besser zu organisieren.	3	2,2±1,4	2,0±1,2	0,180 ^3^	
* * ... bin ich reifer geworden.	4	1,8±1,5	1,7±1,4	0,396	
* ... ist meine Lebenseinstellung positiver geworden.*	5	1,8±1,4	1,4±1,2	**0,002**	**0,37**
* * ... bin ich verantwortungsbewusster geworden.	6	2,0±1,5	1,9±1,4	0,660	
* ... bin ich geduldiger geworden.*	7	2,2±1,4	1,8±1,2	**0,016**	**0,29**
* ... bin ich eine verständnisvollere Person geworden.*	8	2,5±1,2	1,9±1,2	**<0,001**	**0,47**
* ... ist mir deutlicher geworden, welche Werte mir in meinem Leben wichtig sind.*	9	3,0±1,2	2,6±1,2	**0,006**	**0,33**
* ... hat mein Leben mehr Sinn erhalten.*	10	2,0±1,4	1,4±1,2	**<0,001**	**0,49**
* * ... ist der Zusammenhalt in der Familie bzw. mit Freunden oder Bekannten gestärkt worden.	11	2,0±1,4	1,8±1,3	0,194	
* ... ist mein Umgang mit anderen Menschen sicherer geworden.*	12	2,0±1,4	1,5±1,2	**0,002**	**0,37**
* ... habe ich mehr Wertschätzung von anderen erfahren.*	13	2,1±1,4	1,8±1,2	**0,016**	**0,29**
* * ... habe ich viel dazu gelernt.	14	2,7±1,4	2,4±1,2	0,152	

**Anmerkungen.** BBCS=Benefits of Being a Caregiver Scale (Spannweite 0–56, fünfstufige Skala von 0 [stimme überhaupt nicht zu] bis 4 [stimme voll zu]), HPS-k=Kurzversion der Häuslichen Pflege-Skala (Spannweite 0–30, vierstufige Likert-Skala von 0 [stimme überhaupt nicht zu] bis 3 [stimme voll zu]). Signifikante *p* -Werte sind in Fettdruck und die zugehörige Variable in kursiv dargestellt;

^1^ Die dargestellte *p* -Werte sind vor dem Hintergrund des explorativen Charakters der Studie zu interpretieren;

^2^ Cohen’s *d* (bei negativem Wert wird der Betrag | *d* | berichtet);

^3^ Verwendung des Welch-Tests aufgrund von Varianzinhomogenität (sign. Levene-Test).

### Weitere Instrumente

Zusätzlich wurden weitere pflegerelevante Aspekte, wie die ***physische und psychische Gesundheit*** ( *Short-Form-Health-Survey* 28 ), die ***aktuelle Beziehungsqualität*** zwischen pZ bzw. pA und der pP 29 , die ***Zufriedenheit mit der aktuellen Pflegesituation*** (CarerQol-Fragebogen 30 ) und das ***Zurechtkommen mit der Pflegesituation*** eingeschätzt. Diese werden in Tab. 3 näher erläutert.

**Table:** **Tab. 3** Unterschiede zwischen pflegenden Zugehörigen und Angehörigen bezüglich weiterer pflegerelevanter Aspekte.

Variable	Zugehörige ( *N=* 71)	Angehörige ( *N=* 2856)	*p* ^1^	Effektstärke ^ 2^
Physisches Gesundheitsempfinden (Spannweite 0–100), *M* ± *SD*	56,4±24,8	54,9±25,1	0,628	
Psychisches Gesundheitsempfinden (Spannweite 0–100), *M* ± *SD*	58,5±30,3	49,4±27,3	**0,014** ^3^	**0,34**
Beziehungsqualität zur gepflegten Person, *N* (%)				
neutral/negativ	18 (25,4)	1124 (39,4)	**0,017**	**0,04**
positiv	53 (74,6)	1732 (60,6)		
Zufriedenheit mit der Pflegesituation (Spannweite 0–10), *M* ± *SD*	5,7±2,6	4,9±2,4	**0,003**	**0,35**
Zurechtkommen mit der Pflegesituation (Spannweite 1–10), *M* ± *SD*	7,2±2,0	6,6±2,1	**0,017**	**0,29**

**Anmerkungen.** Physisches Gesundheitsempfinden (visuelle Analogskala [ *schlecht* bis *ausgezeichnet* ]): „Wie würden Sie Ihre körperliche Gesundheit beschreiben?“, Psychisches Gesundheitsempfinden (visuelle Analogskala [ *schlecht* bis *ausgezeichnet])* : „Wie würden Sie Ihre seelische Gesundheit beschreiben?“, Beziehungsqualität zur gepflegten Person (dreistufige Skala [ *positiv, neutral, negativ* ], wobei die Stufen *neutral* und *negativ* zusammengefasst wurden): „Wie schätzen Sie aktuell die Qualität der Beziehung zu der von Ihnen unterstützten, betreuten oder gepflegten Person ein?“, Zufriedenheit mit der Pflegesituation (visuelle Analogskala [ *vollkommen unzufrieden* bis *vollkommen zufrieden* ]): „Kreuzen Sie bitte in der nachfolgenden Skala an, wie zufrieden Sie sich im Moment in Ihrer Unterstützungs-/Betreuungs-/Pflegesituation fühlen.“, Zurechtkommen mit der Pflegesituation (10-stufige Skala [ *gelingt mir gar nicht* bis *gelingt mir vollständig* ]): „ Wie schätzen Sie aktuell Ihre Möglichkeiten ein, mit der Pflege zurechtzukommen?“;

^1^ Die dargestellte *p* -Werte sind vor dem Hintergrund des explorativen Charakters der Studie zu interpretieren;

^2^ Cramer’s V für kategoriale Variablen, Cohen’s d (Betrag |d| bei negativem Wert) für metrische Variablen;

^3^ Verwendung des Welch-Tests aufgrund von Varianzinhomogenität (sign. Levene-Test).

### Statistische Analyse

Im Rahmen der Hauptanalyse fand ein Vergleich zwischen pZ und pA bezüglich der im Durchschnitt erfahrenen objektiven und subjektiven Belastungen sowie des Zugewinnerlebens durch die häusliche Pflege statt. Mittels Zusatzanalysen wurden auf Itemebene die spezifisch erlebten Belastungen und Zugewinne sowie weitere pflegerelevante Aspekte untersucht, um Aussagen über deren konkrete inhaltliche Bedeutsamkeit treffen zu können und pZ erstmals umfassend zu untersuchen.

Für die statistischen Analysen wurde IBM SPSS Version 28 verwendet (α=0,05). Aufgrund des explorativen, hypothesengenerierenden Charakters dieser Studie konnte auf eine Alpha-Fehler-Korrektur verzichtet werden 31 .

Zur Schätzung fehlender Werte wurde für metrische Variablen der Expectation-Maximization-Algorithmus und für kategoriale Variablen eine Modusimputation verwendet. Der Großteil der Variablen besaß weniger als 5% fehlende Werte, wobei die metrische Variable *Beaufsichtigung in Stunden/Tag* mit 21,9% und die kategoriale Variable *Geschlecht der gepflegten Person* mit 5,3% die höchste Anzahl an fehlenden Werten aufwiesen.

Zusätzlich zu deskriptiven Parametern wurde für die inferenzstatistische Testung des Unterschieds zwischen pZ und pA für metrische Variablen der *t* -Test für unabhängige Stichproben bzw. bei Varianzinhomogenität der Welch-Test sowie bei Vorhandensein extremer Ausreißer (>2,5-facher Interquartilsabstand) der nichtparametrische Mann-Withney-U-Test berechnet. Für kategoriale Variablen wurde der χ ^2^ -Test bzw. der exakte Test nach Fisher (Zellhäufigkeiten<5 Fälle) verwendet. Effektstärken wurden mittels Cramer’s V und Cohen’s *d* berichtet.

## Ergebnisse

### Allgemeine Charakteristika

Von den *N* =2927 Befragten waren *n=* 71 (2,4%) pZ und *n=* 2856 (97,6%) pA. Die Verwandtschafts- bzw. Beziehungsverhältnisse sind in **Online-Zusatzabbildung S1** dargestellt. Es zeigten sich keine relevanten Unterschiede zwischen pZ und pA hinsichtlich der allgemeinen Charakteristika ( Tab. 1 ), mit der Ausnahme, dass pZ signifikant seltener mit der pP zusammenwohnten als pA.

**Abb. 1:**
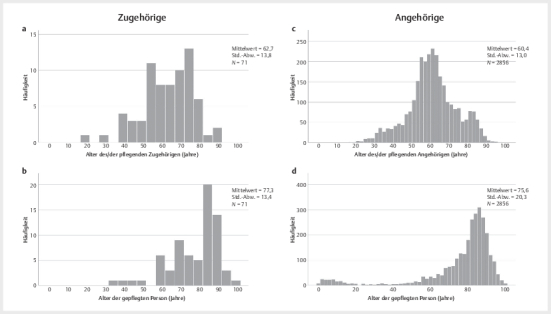
Altersverteilung der pflegenden Zu- ( **a** ) und Angehörigen ( **c** ) sowie deren gepflegter Personen ( **b, d** ).

Bei beiden Gruppen zeigte sich eine Altersverteilung, die vom jungen bis in das hochaltrige Erwachsenenalter reichte ( Abb. 1 ). Die Altersverteilung der pA zeigte ihren Peak im höheren Erwachsenenalter, diejenige der pZ zeigte je einen Peak im Erwachsenen- und einen im höheren Erwachsenenalter. Beide Altersverteilungen der pP zeigten Peaks im hochaltrigen Bereich. Anders als die pA pflegten pZ keine Kinder, Jugendlichen oder junge Erwachsene unter 30 Jahren.

### Belastungen und Zugewinne

**Abb. 2:**
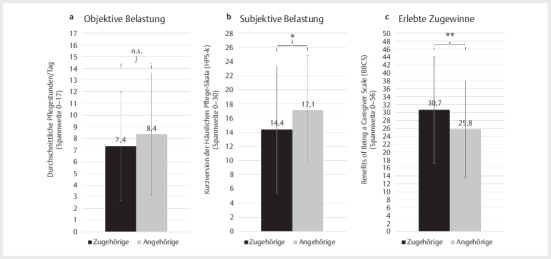
Unterschiede zwischen pflegenden Zugehörigen und Angehörigen für ( **a** ) die objektive Belastung, ( **b** ) die subjektiv wahrgenommene Belastung und ( **c** ) die erlebten Zugewinne durch die häusliche Pflege; bei ( **a** ) und ( **b** ) wurde der Welch-Test aufgrund gegebener Varianzinhomogenität (sign. Levene-Test) berechnet; *p<0,05; **p<0,001; die dargestellten p-Werte sind vor dem Hintergrund des explorativen Charakters der Studie zu interpretieren.

Es zeigte sich, dass sich pZ und pA nicht signifikant bezüglich der objektiven Belastung ( *t* (74,45)=1,80, *p* =0,077) unterschieden. Subjektiv nahmen pZ ein signifikant geringeres Belastungsempfinden wahr ( *t* (72,56)=2,57, *p* =0,012, *d* =0,36) und erlebten zudem signifikant mehr Zugewinne ( *t* (2925)=−3,37, *p* <0,001, | *d|* =0,41) durch die häusliche Pflegesituation als pA (s. Abb. 2 ).

### Zusatzanalysen auf Itemebene: Spezifisch erlebte Belastungen und Zugewinne

Hinsichtlich der spezifisch erlebten Belastungen schätzten pZ ihre körperliche Erschöpfung (Variable 2), die Depersonalisationssymptomatik (Variable 4), die Einschränkung der eigenen Gesundheit (Variable 6) sowie die der Lebenszufriedenheit (Variable 1) signifikant niedriger ein als pA. Bei den erlebten Zugewinnen zeigte sich, dass pZ im Vergleich zu pA signifikant mehr Sicherheit im Umgang mit anderen (Variable 12), vermehrtes Sinnerleben durch die Pflegetätigkeit (Variable 10), häufiger von einer Neuausrichtung der eigenen Wertevorstellungen (Variable 9) und von einer Verbesserung der Beziehungsqualität zur pP (Variable 2) berichteten. Daneben gaben sie signifikant häufiger an, eine positivere Lebenseinstellung zu zeigen (Variable 5), geduldiger sowie verständnisvoller gegenüber der pP zu sein (Variable 7, 8) und mehr Wertschätzung von anderen zu erfahren (Variable 13) (s. Tab. 2 ).

### Analyse weiterer pflegerelevanter Aspekte

Die Analysen (s. Tab. 3 ) zeigten, dass pZ ihre psychische Gesundheit signifikant besser einschätzten als pA ( *t* (72,84)=−2,52, *p* =0,014), jedoch nicht ihre physische Gesundheit ( *t* (2925)=−0,49, *p* =0,628). Die Beziehungsqualität zur pP wurde von den pZ tendenziell positiver eingeschätzt ( *χ²* (1)=5,71, *p* =0,017). pZ gaben an, mit der Pflegesituation zufriedener zu sein ( *t* (2925)=−2,92, *p* =0,003) und besser mit ihr zurechtzukommen ( *t* (2925)=−2,39, *p* =0,017).

## Diskussion

Ziel der Studie war es, die Situation der pZ empirisch-quantitativ zu untersuchen. Es zeigte sich der Haupttrend, dass pZ bei fast gleichen allgemeinen Charakteristika und gleicher objektiver Belastung die häusliche Pflegesituation als weniger belastend und vielmehr als persönlichen Zugewinn wahrnahmen und ihre psychische Gesundheit positiver einschätzten als pA.

Die pZ und pA unterschieden sich nicht hinsichtlich relevanter soziodemographischer Charakteristika, sodass von ähnlichen Merkmalen ausgegangen werden kann. 2,4% der informellen Pflegepersonen waren pZ. Diese geringe Anzahl an pZ deckt sich mit den Erkenntnissen der Literatur, wonach ca. 2% pZ sind 11 . Dass diese Gruppe in Zukunft größer und wichtiger werden wird, zeigen Ergebnisse, wonach pP, denen ein räumlich verfügbares Netzwerk verwandter Personen fehlt, schon jetzt verstärkt auf nicht-verwandte Personen zur Bewältigung ihres Alltags zurückgreifen 6 7 8 20 . Die Gründe für die Nicht-Verfügbarkeit von pA sind vielfältig und inkludieren z.B. häufigere Kinderlosigkeit bzw. die räumliche Trennung von den Kindern und anderen verwandten Personen 6 7 . Die Altersverteilung zeigte, dass pZ sowohl im mittleren als auch im höheren Erwachsenenalter Pflege leisteten. Dies deckt sich mit der Literatur, wonach Freund*innen ein höheres Alter aufwiesen und entsprechend eher Altersgenossen pflegten, wohingegen Nachbar*innen tendenziell jünger waren und im mittleren Erwachsenenalter pflegten 12 . Es zeigte sich zudem, dass pZ anders als pA keine Kinder, Jugendlichen oder jungen Erwachsenen unter 30 Jahren pflegten. Dies erscheint schlüssig vor dem Hintergrund berichteter Pflegemotive 11 , wonach die Pflege des leiblichen Kindes als selbstverständlich erachtet wird und aus dem Gefühl der Zuneigung heraus erfolgt, sodass pZ seltener in Erscheinung treten. Die Ergebnisse aus der Literatur über die Art der geleisteten Unterstützung, wonach pZ mehr Zeit für IADLs und Beaufsichtigung der pP und weniger für ADLs aufbringen 6 , konnten repliziert werden.

Es konnte gezeigt werden, dass sich pZ und pA nicht hinsichtlich der objektiven Pflegebelastung unterschieden, pZ das subjektive Belastungserleben jedoch niedriger einschätzten. Die Zusatzanalysen auf Itemebene der spezifisch erlebten Belastungen zeigten, dass pZ die eigene körperliche Erschöpfung und Depersonalisationssymptomatik niedriger einschätzten. Erschöpfungs- und Depersonalisationssymptome gelten als klinische Kernkriterien des Burnouts 32 , das für pA eine häufige Folge der häuslichen Pflege darstellt 33 . Entsprechend kann angenommen werden, dass pZ ein geringeres Burnout-Risiko haben könnten als pA. Unterstützt wird diese Annahme auch durch die Ergebnisse der Zusatzanalysen auf Itemebene der Variablen sechs der HPS-k sowie zum Gesundheitserleben, wonach pZ die eigene psychische Gesundheit ebenfalls signifikant positiver einschätzten als pA.

Es wurde ein potentiell existierender „Puffereffekt“ des Zugewinnerlebens auf die psychische Gesundheit für pA berichtet 25 . Die Ergebnisse der vorliegenden Studie sind ein Hinweis dafür, dass auch für pZ dieser Effekt existieren könnte. Die pZ nahmen bei gleicher objektiver Belastung und geringerem subjektiven Belastungserleben signifikant mehr positive Pflegeaspekte wahr und schätzten ihre psychische Gesundheit signifikant positiver ein. Die Zusatzanalysen auf Itemebene zeigten, dass pZ von einer Vielzahl unterschiedlicher Aspekte durch die Pflege profitieren (Sicherheit im Umgang mit anderen, vermehrte Wertschätzung durch Außenstehende, vermehrtes Sinnerleben durch die Pflegetätigkeit, Neuausrichtung eigener Wertevorstellungen, geduldigerer und verständnisvollerer Umgang mit der pP). Auch pP könnten von der Pflege durch pZ profitieren, da sich im Vergleich zu pA die Beziehungsqualität zum/zur pZ verbesserte. Die Forschung mit pA zeigt, dass eine als positiv erlebte Beziehung das subjektive Belastungserleben absenken 34 und Einfluss nehmen kann auf das Risiko, eine psychische Symptomatik auszubilden 35 36 . Zukünftige Forschung sollte dies auch für pZ prüfen.

Zur Beantwortung der Frage, warum pZ durch die Pflege einer pP nicht nur geringer belastet sind, sondern von dieser zu profitieren scheinen, könnte das Pflegemotiv eine wichtige Rolle spielen. Während die Pflege aus Zuneigung aus einer emotionalen Verbindung zur pP heraus erwächst, finden sich bei der Pflege aus Verpflichtung verschiedene Submotive wie z.B. das Gefühl der moralischen Verpflichtung 37 . So geht mit dem Zuneigungsmotiv eine größere Selbstbestimmtheit und Freiwilligkeit bei der Entscheidung für die Pflege einher, was negative Assoziationen zum subjektiven Belastungserleben erklären könnte. Dies lässt sich auf die Gruppe der pZ übertragen. Bisherige Forschung zeigt, dass die Bereitschaft zur Pflege von pZ trotz der fehlenden verwandtschaftlichen Verbindung mit Überzeugungen einhergeht 16 , die sich als Pflege aus Zuneigung definieren lässt und mit einem geringeren Belastungserleben assoziiert sein könnte 37 . So erfolgt die Pflege meist aus einer positiven Motivation heraus 15 . pZ repräsentieren Personen, deren Natur es ist, zu helfen 23 . Auch Mitleid mit den Alleinlebenden und Aktionismus wurden als Motive angegeben 23 . Somit ist die Pflege durch pZ geprägt durch ein hohes Engagement und eine hohe Beziehungsqualität zwischen pZ und pP 16 . Die Konsequenzen der Pflege zeigen sich für pZ vermehrt positiv und inkludieren u.a. das Gefühl von Zufriedenheit 23 und das Empfinden des persönlichen Gewinns einer Freundschaft 15 . Folglich kann davon ausgegangen werden, dass das Motiv „Pflege aus Zuneigung“ das geringere Belastungserleben und das vermehrte Zugewinnerleben der pZ teilweise erklären könnte.

Vor dem Hintergrund des geringen Belastungs- und vermehrten Zugewinnerleben erscheint das Ergebnis der signifikant höheren Zufriedenheit der pZ mit der Pflegesituation sowie das bessere Zurechtkommen mit dieser als schlüssig. Die Zufriedenheit und das Zurechtkommen mit der Pflegesituation bilden zudem eine wesentliche Grundlage für eine möglichst lange Aufrechterhaltung der Pflege bis zum Lebensende der pP, wenn hohe Belastungen für die informellen Pflegepersonen besonders häufig auftreten 6 . Somit bilden pZ eine bisher bei weitem noch nicht ausgeschöpfte Ressource, um den möglichst langen Verbleib der pP im häuslichen Umfeld zu ermöglichen, deren Wunsch nach Autonomie zu begegnen und die häusliche Pflege zu stärken. Mit konkreten Maßnahmen, wie der Entwicklung spezifisch auf die Bedarfe und Bedürfnisse der pZ zugeschnittener Entlastungs- und Unterstützungsangebote sollte versucht werden, diese Ressource gezielt zu stärken.

## Stärken und Limitationen

Dem Kenntnisstand der Autor*innen zufolge ist die vorliegende Studie die erste, die pZ über die allgemeinen Charakteristika hinaus untersucht, mehrere bedeutsame Gesundheits- und Versorgungsvariablen erfasst und Unterschiede zu den pA aufzeigt. Damit wurde begonnen, die Forschungslücke „pZ“ zu schließen. Die umfangreiche Stichprobe ist repräsentativ für die pZ und pA der gesetzlich Pflegeversicherten mit Erst- oder Folgeantrag auf Pflegegradeinstufung in Bayern.

Limitierend wirkt der explorative Charakter der Analysen, da aufgrund begrenzter vorheriger Forschung nicht hypothesengeleitet untersucht werden konnte. Alle Informationen beruhen auf Selbstberichten der Befragten und unterliegen somit den bekannten Antworttendenzen. Die Rücklaufquote ist – möglicherweise pandemiebedingt – als niedrig einzustufen. Dieser Grund und die Befragungsregion Bayern limitieren die nationale bzw. internationale Übertragung der Ergebnisse.

## Fazit für die Praxis

- pZ profitieren vielfach von der Unterstützung, Betreuung oder Pflege einer nicht-verwandten Person: Sie erleben im Vergleich zu pA weniger subjektive Belastung, eine höhere psychische Gesundheit und mehr persönliche Zugewinne.
- pZ stellen somit eine wesentliche, noch nicht voll ausgeschöpfte Ressource für die zukünftige Stärkung der häuslichen Pflege dar.
- Die Vorteile, die pZ im Rahmen der Pflege einer pP erfahren, sollten gezielt genutzt werden, um zukünftig vermehrt pZ zur Stärkung der häuslichen Pflege zu gewinnen.
- Die Stärkung und Unterstützung der pZ ist mit Blick auf die zukünftig steigende Zahl an pP infolge des demografischen Wandels sowie die gleichzeitig größer werdende Problematik des Fachkräftemangels im Gesundheitssektor wesentlich.

## Fördermittel

Reinhard Frank-Stiftung — Die Studie ‚Fortschritt in der häuslichen Pflege (Progression in home care; ProCare)‘ wurde durch die Reinhard Frank-Stiftung gefördert.

## Notes

Abkürzungen **ADLs** Aktivitäten des täglichen Lebens
**BBCS** Benefits of Being a Caregiver-Skala
**HPS-k** Häusliche Pflegeskala – Kurzversion
**IADLs** Instrumentelle Aktivitäten des täglichen Lebens
**pA** pflegende*r Angehörige*r (Singular und Plural)
**pP** pflegebedürftige Person (Singular und Plural)
**pZ** pflegende*r Zugehörige*r (Singular und Plural)

## Zusätzliches Material

Zusätzliches Material Zusätzliches Material
