# Supplementary material for: When non-kin caregivers provide home care: additional burden or personal benefit for friends, neighbours, and acquaintances? – Findings from an exploratory cross-sectional study
Source: Gesundheitswesen. 2026 Feb 2;88(6):395–403. [Article in German] doi: 10.1055/a-2736-6425 (PMC13246226; doi:10.1055/a-2736-6425)
Supplement: Supplementary file 1 — Zusätzliches Material [file 10-1055-a-2736-6425-gesu-2025-02-2226-oa.pdf]

Online-Zusatzabbildung S1: Verwandtschafts-/Beziehungsverhältnis zwischen (a) Zugehörigen und pflegebedürftiger Person („Sonstiges" inkludiert pflegebedürftige Personen, bei denen weniger enge Beziehungen zum/zur Zugehörigen bestehen [„entfernte Bekannte"], z. B. Expartner*innen der Ehefrau/des Ehemannes) und (b) Angehörigen und pflegebedürftiger Person

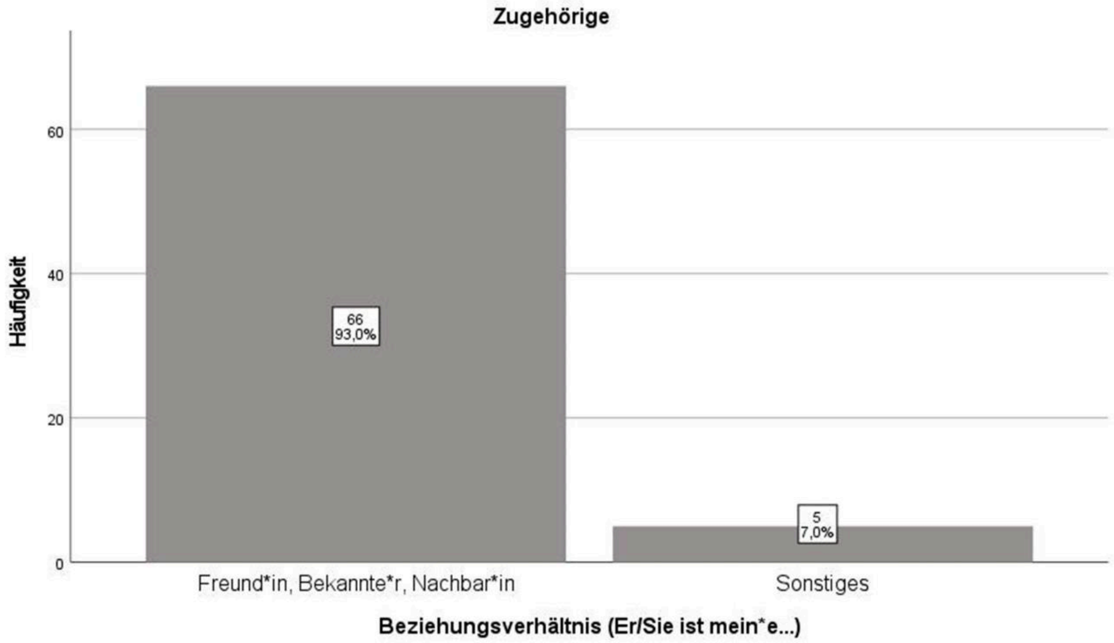

(a)

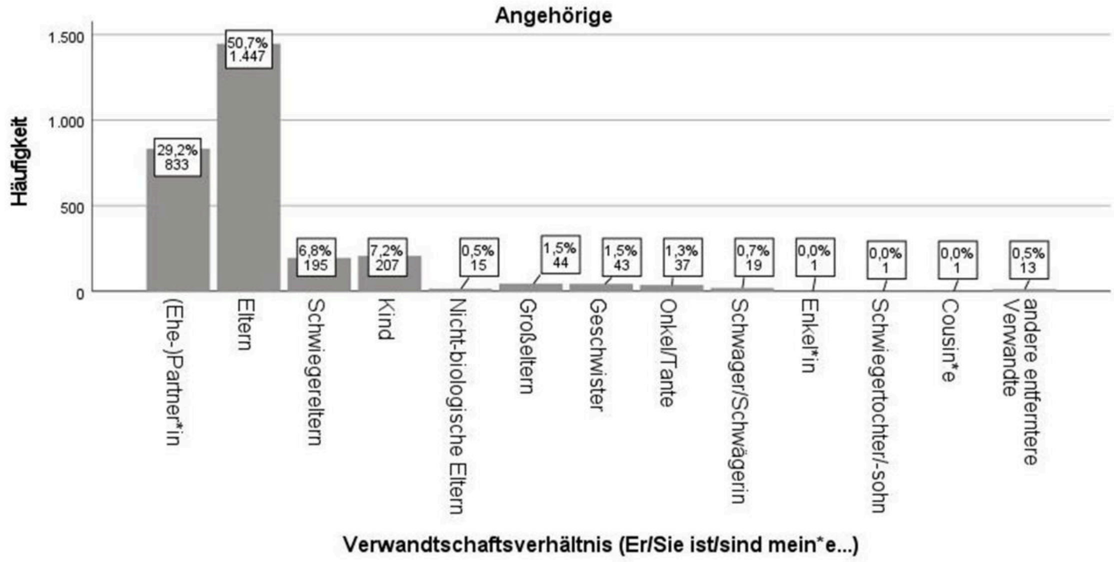

(b)
